# Smart Strategies for Improving Electric Vehicle Battery Performance and Efficiency

**DOI:** 10.1038/s41598-025-25987-1

**Published:** 2025-11-26

**Authors:** Swathi Tangi, Ayush Vatsa, Akshat Opam, Praveen Kumar Bonthagorla, D. N. Gaonkar

**Affiliations:** 1 Department of Electrical and Electronics Engineering, Manipal Institute of Technology, Manipal Academy of Higher Education, Manipal, Karnataka 576104 India; 2 Department of Electrical and Electronics Engineering, National Institute of Technology Karnataka, Surathkal, Karnataka 575025 India

**Keywords:** Electric vehicles (EVs), Range estimation, Machine learning, Ensemble learning, Optimal driving conditions, Engineering, Electrical and electronic engineering

## Abstract

The increasing demand for Electric Vehicles (EVs) necessitates accurate range prediction and optimization of driving parameters to address range anxiety and improve user experience. This study proposes a machine learning-based framework for predicting EV range, optimum acceleration, and velocity using a synthetically generated dataset of 2,000 samples designed to reflect real-world driving scenarios. Four models—Random Forest (RF), Extra Trees (ET), Linear Regression (LR), and Long Short-Term Memory (LSTM)—were evaluated individually and in ensemble combinations. To ensure statistical reliability, all models were trained and tested over ten independent runs with randomized data partitions, and the results were reported as average performance with standard deviations. The ensembles consistently outperformed individual models, with the full ensemble (RF + ET + LSTM + LR) achieving the most robust performance across all metrics (MAE, MSE, and R²). Furthermore, a real-time web application was developed using the trained models to dynamically estimate driving parameters. The findings highlight the potential of integrating AI-driven predictive modelling into EV systems to support efficient driving behaviour and energy management.

## Introduction

Despite challenges like limited range and high battery costs, recent EV research focuses on energy integration, battery improvements, infrastructure, and adoption strategies. These efforts, driven by international collaboration, aim to overcome barriers and position EVs as key solutions for climate change and urban air quality^1^. This integrative assessment provides insights for investors and policymakers by analysing global EV demand, technological advancements, energy storage, charging methods, and smart city development. It aims to support informed decisions in the evolving electric mobility landscape^2^. The success of the electric vehicle industry depends on technological innovation, strong policy support, and environmental sustainability. Key enablers include government backing, sustainable development practices, and widespread charging infrastructure^3^. To promote widespread adoption and enhance EV efficiency and convenience, battery technology development must tackle challenges such as high cost, range anxiety, charging time, lifespan, performance, weight, and safety^4^. Renewable energy sources are emerging as a viable alternative to fossil fuels, driven by climate change concerns, depleting fossil reserves, and escalating energy and transportation costs^5^. The transport sector emits major greenhouse gases, but EV adoption can significantly cut emissions and support the shift to renewables^6^.

The Random Forest model outperformed other machine learning models in forecasting EV battery charging cycles. The proposed application leverages these models to support accurate maintenance planning and energy management decisions^7^. To enhance prediction accuracy for EV session duration and energy consumption, the study integrates historical charging data with weather, traffic, and local events. The ensemble learning model delivers the most accurate and reliable outcomes^8^. The study demonstrates the superior performance of Automated Machine Learning (AutoML) by a hybrid model to estimate EV sales, utilizing a dataset comprising 357 newly manufactured cars in the U.S. between 2014 and 2020^9^. A study exploring battery reuse in Colombia forecasts electric vehicle sales using a hybrid LSTM and Convolutional LSTM model, achieving superior performance with an acceptable error margin of 3.5%, outperforming other models^10^. This study addresses issues and offers insights for researchers and practitioners by investigating the application of predictive machine learning approaches to improve the performance of electric vehicle (EV) batteries^11^.

The article proposes a centralized routing approach for Vehicular Ad hoc Networks (VANETs) that leverages AI-powered mobility predictions through an SDN controller, optimizing routing paths, reducing transmission delays, and adapting to dynamic network conditions^12^. The research proposes an ensemble learning and voltage reconstruction framework for estimating lithium-ion battery State of Health (SOH) from random charging, achieving a 3.42% error^13^. The paper proposes a deep learning-based driver drowsiness detection system with high accuracy, showing strong potential for intelligent transport applications^14^. The paper presents a machine learning-based rotor position estimator for interior permanent magnet synchronous motors, demonstrating cost-efficiency and robustness across various speeds and plans to address limitations^15^. The study uses a hybrid machine-learning approach with Extreme Gradient Boosting (XGBoost), Light Gradient-Boosting Machine (LightGBM), and an anchor-based method to forecast EV driving range, improving accuracy with motor/battery energy, driving patterns, and temperature data^16^. This research presents a machine-learning technique for online Li-ion battery state-of-health estimation in EVs, achieving under 2% error using partial charging curves and similarity factors^17^. To assist researchers in creating efficient overtaking solutions, the review paper assesses several computer vision techniques for augmented intelligence in autonomous vehicles (AVs) and their performance on various datasets^18^. The paper addresses BEV drivers’ challenges with limited range and charging infrastructure, suggesting probabilistic prediction models and federated learning for accurate energy demand and driving range predictions^19^. The article presents a technique that uses active power measured at the charging socket to estimate the charge level of onboard batteries in light-EVs^20^. The article discusses RFID tags in IoT infrastructure, highlighting the need for accurate prediction models and further research on factors like vehicle speed and windshield tint^21^. The paper explores machine learning’s potential in high-mobility vehicular networks, highlighting its ability to optimize performance through dynamic environments and advanced sensor data generation^22^. The study examines weaknesses in defensive distillation-based mitigation techniques and deep learning-based channel estimate models for adversarial attacks in Next Generation wireless networks, proving their efficacy^23^. To enhance energy consumption and recharge schedules, the study presents a unique algorithm for assessing the level of charge in EVs that uses meta-experience replay and continuous learning^24^.

The paper presents a multi-sensor fusion estimator for EV speed, combining IMU and Global Positioning System- Beidou (GPS-BD) positioning modules, with high accuracy shown through hardware-in-the-loop testing^25^. With an ideal driving speed of 58 km/h, the research proposes an online remaining driving range (RDR) prediction model for battery EVs that reveals a 39% higher ECR and 30% reduction in winter^26^. The study uses data-driven techniques to analyze the impact of EV parameters on range, revealing strong correlations between battery capacity, top speed, curb weight, and acceleration^27^. To overcome data sparsity and noise problems and provide better performance than conventional techniques, the research presents a deep learning framework for predicting land vehicle ego-velocity using Frequency Modulated Continuous Wave (FMCW) automobile radars^28^. The study investigates how the feasibility of battery EVs (BEVs) is affected by accurate range assessment and discovers that altering the cumulative prospect theory parameter can make BEVs more feasible^29^. The research introduces a stacked extended LSTM network to accurately predict lithium iron phosphate battery dynamics and estimate SOC from current, voltage, and temperature^30^. Table 1 outlines the comparative evaluation of previous research efforts.

**Table 1:** Comparative review of existing literature.

Ref.	Focus area	AI/ML used	Novelty/ strength	Application domain
^31^	V2G integration for grid support	Optimization	Multi-objective grid + user-centric	Smart grid, power systems
^32^	Battery safety & performance	CNN, FNN, PSO	Hybrid DL methods for prediction	Battery safety & BMS
^33^	Smart BMS review (2010–2020)	Review-based	Bibliometric + technical analysis	EV battery technologies
^34^	EV tech trends (1990–2022)	Bibliometrics	Holistic policy-tech-infra synergy	EV policy & V2X development
^35^	BMS optimization with RL	Q-learning	Real-time dynamic optimization	BMS performance & cost
^11^	Battery predictive modeling	ML + OR	Multi-ML approach + real-time insights	Predictive BMS for EVs
^36^	Optimal EV Charging & Grid Impact	AI-driven scheduling and pattern-based optimization	Large-scale adoption analysis (1.5 M–5 M EVs), duck curve smoothing, peak demand reduction up to 34%	Grid-level charging optimization
^37^	Dynamic Charging Optimization	Dynamic scheduling algorithm (Proof of Need – PoN)	Priority index using SoC, charger power, user preferences, substation capacity; 40.8% demand reduction	Distribution grid & household-level EV charging
^38^	EV Powertrain & Range modeling	Analytical & numerical modeling (review)	Comprehensive review of analytical models, driving cycles, and powertrain configurations	EV design & simulation (powertrain optimization)
^39^	Real-time range prediction with battery capacity index	BiLSTM–KAN, SHAP for interpretability	Introduces Battery Capacity Index (BCI) for aging effects; captures real-time driving + battery health	Vehicle-level range prediction
^40^	Deep learning range prediction	Deep Neural Network (DNN) with RMSProp optimizer	Achieved very high accuracy (R² = 0.99, MAE = 6.81 km); optimizer benchmarking	Real-world driving range estimation
^41^	Energy consumption prediction with ensembles	KNN + Tree-based ensemble, Optuna, PSO, GridSearch	Hybrid stacking ensemble; hyperparameter tuning comparison; R² = 0.96	EV energy consumption forecasting
^42^	Campus case study – driving dynamics	Extra Trees, CatBoost, LightGBM, XGBoost	Experimental dataset (~ 2 km route); slope, load, and driving dynamics quantified; Extra Trees best performer	Route-level range estimation & battery management

Existing studies on AI/ML sin EVs largely focus on charging, driver behaviour, or battery degradation at subsystem or grid levels. Few works address integrated vehicle-level range estimation with optimal driving parameters. This study introduces an ensemble ML framework that jointly estimates EV range and predicts optimum acceleration and velocity for holistic performance optimization.

The key contributions of this proposed work are as follows:

1. Developed a machine learning framework to predict EV range, optimum velocity, and acceleration.
2. Created a realistic custom dataset based on EV parameters due to lack of public data.
3. Evaluated and compared four models: Random Forest, Extra Trees, LSTM, and Linear Regression.
4. Achieved improved accuracy using ensemble techniques, with the full ensemble performing best.
5. Deployed the model in a web-based application for real-time prediction and user interaction.

## Methodology

### General methodology of proposed work

**Fig. 1:**
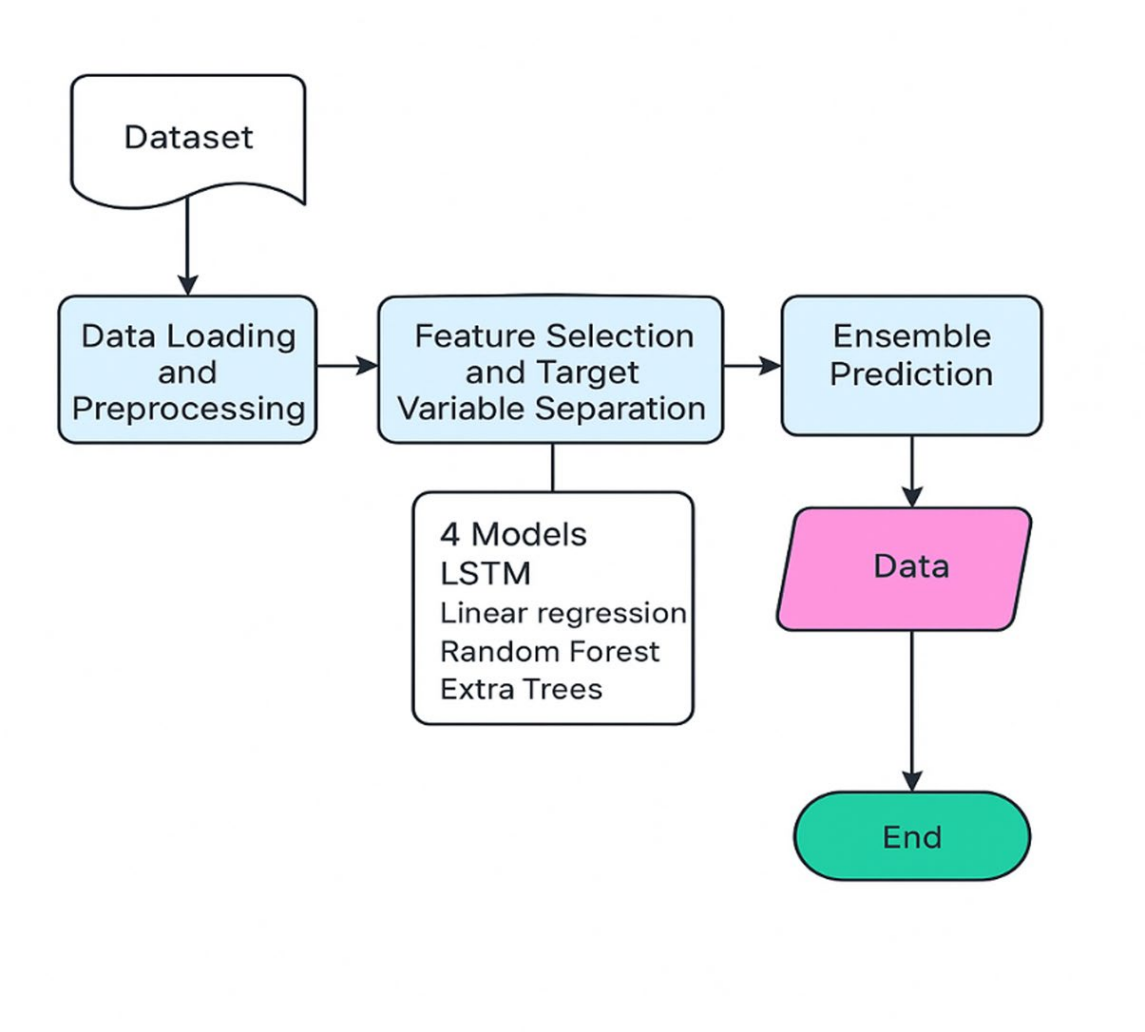
General Methodology of Proposed Work.

The methodology involves a systematic approach to use different models to predict range and optimum driving condition (acceleration and velocity). As shown in Fig. 1, it starts with dataset gathering and pre-processing of dataset to fit the models and to be used in the training and testing of the models. The dataset has been created using Python to mimic real EV working situations. This step ensures that the data is in a suitable format for subsequent analysis and model training.

Following the data processing, the feature and targets to the models are defined in the python code so that the model knows to calculate what on based of what. The features include speed, acceleration, elevation change, temperature, weather conditions, HVAC usage, battery health, state of charge, and driving mode. While training for different models, namely for range, optimum acceleration and velocity, the prescribed value using calculation ranging from complex equations to basic Newton’s models to get the target values are created. Based on these target values in different models, the model is adapted.

Four different models are chosen to train and test the data namely Random Forest Regressor, Extra Trees Regressor, LSTM (Long short-term memory), and Linear Regression. These models are taken for their good proximity with the results involving linear results. Having the training done in four different models gives a chance to compare the results of each model individually and provides the opportunity to ensemble results in all combinations possible and compare which is most relevant and helps in choosing optimal results. The performance of these models is assessed using metrics like Mean Absolute Error (MAE), Mean Squared Error (MSE), and R-squared (R²).

### Linear regression (LR)

Mathematical Equation: Linear regression models the relationship between an input feature and the target variable using a linear equation. For simple linear regression with one input, the equation is:

1 $$\:y={\beta\:}_{0}+{\beta\:}_{1}\times\:x\:\:\:\:$$

Where $\:y$ is the predicted target variable, $\:x$ is the input feature, and $\:{\beta\:}_{0}$ and $\:{\beta\:}_{1}$ are the intercept and slope coefficients, respectively. In multiple linear regression with multiple input features, the equation extends to include multiple coefficients. Algorithmic Approach: Linear regression minimizes the sum of the squared differences between the observed and predicted target values by adjusting the coefficients $\:{\beta\:}_{0},{\beta\:}_{1},\dots\:,{\beta\:}_{n}$ using techniques like Ordinary Least Squares (OLS) or gradient descent.

### Random forest (RF) and extra trees (ET)

Random Forest and Extra Trees are ensemble methods using multiple decision trees. They build trees on random data subsets and feature splits, then aggregate predictions to reduce variance and improve accuracy over single decision trees.

2 $$\:f\left(y\right)=\frac{1}{N}\sum\:_{n=1}^{N}\:{f}_{M}\left(y\right)$$

Where $\:{f}_{M}$is the aggregated prediction from the Random Forest ensemble consisting of M decision trees, and N is the number of trees. The projected value at the data point y for the i-th tree in the family is represented as $\:{f}_{M}$(y; X_i_, D_M_), where X_i_…….X_N_ are the independent input samples (feature vectors) used to construct each tree, distributed independently of D_M_ and in the same manner as the generic random variable X_*i*_. $\:y$ : the target variable corresponding to each input sample. M: the total number of decision trees in the ensemble. Extra Trees (Extremely Randomized Trees) are like Random Forest but add more randomness by choosing random thresholds for splits instead of selecting the optimal ones.

### Long short-term memory (LSTM)

LSTM is a recurrent neural network (RNN) variant designed to capture long-term dependencies in sequences. It uses memory cells and gates (input, forget, output) to control information flow, enabling the network to maintain information across long sequences. LSTMs use sequential learning mechanisms to process input sequences and update internal states over time, enabling them to model complex temporal patterns. LSTMs involve a series of mathematical operations to compute activations, update internal states, and produce output predictions. These operations include matrix multiplications, element-wise operations, and activation functions like sigmoid and tanh, but they do not have a single mathematical equation like linear regression.

3 $$\:\left.\begin{array}{ll}{i}_{t}&\:\:=\sigma\:\left({W}_{xi}{x}_{t}+{W}_{hi}{h}_{t-1}+{W}_{ci}{c}_{t-1}+{b}_{i}\right)\\\:{f}_{t}&\:\:=\sigma\:\left({W}_{xf}{x}_{t}+{W}_{hf}{h}_{t-1}+{W}_{cf}{c}_{t-1}+{b}_{f}\right)\\\:{g}_{t}&\:\:=\text{t}\text{a}\text{n}\text{h}\left({W}_{xg}{x}_{t}+{W}_{hg}{h}_{t-1}+{b}_{g}\right)\\\:{c}_{t}&\:\:={f}_{t}\odot\:{c}_{t-1}+{i}_{t}\odot\:{g}_{t}\\\:{o}_{t}&\:\:=\sigma\:\left({W}_{xo}{x}_{t}+{W}_{ho}{h}_{t-1}+{W}_{co}{c}_{t}+{b}_{o}\right)\\\:{h}_{t}&\:\:={o}_{t}\odot\:\text{t}\text{a}\text{n}\text{h}\left({c}_{t}\right)\end{array}\right\}\:\:\:\:\:\:$$

Where, $\:{i}_{t},{f}_{t},{o}_{t}$ are the input, forget, and output gate activations at time step $\:t$, $\:{g}_{t}$ is the candidate cell value at time step $\:t$. $\:{c}_{t}$ is the cell state at time step $\:t$. $\:{h}_{t}$ is the hidden state at time step $\:t$. $\:{x}_{t}$ is the input at time step $\:t$. $\:{W}_{..}$are weight matrices, $\:b$. are bias vectors. $\:\sigma\:$ is the sigmoid function, tanh is the hyperbolic tangent function. $\:\odot\:$ denotes element-wise multiplication.

MAE (Mean Absolute Error) measures the average absolute difference between predicted and actual values—lower MAE indicates better accuracy. MSE (Mean Squared Error) averages the squared prediction errors, penalizing larger errors more heavily and making it sensitive to outliers; it’s also widely used due to its smooth, differentiable nature. R-squared indicates how well the model explains the variance in the target variable, ranging from 0 (no explanation) to 1 (perfect explanation).

4 $$\:MAE=\frac{1}{n}{\sum\:}_{i=1}^{n}\left|\mathcal{Y}i-\widehat{\mathcal{Y}i}\right|\:\:\:\:\:\:\:\:\:\:\:\:\:\:\:\:\:\:\:\:\:\:\:\:\:\:\:\:$$

5 $$\:MSE=\frac{1}{n}{\sum\:}_{i=1}^{n}{\left(\mathcal{Y}i-\widehat{\mathcal{Y}i}\right)}^{2}\:\:\:\:\:\:\:\:\:\:\:\:\:\:\:\:\:\:\:\:\:\:$$

6 $$\:{R}^{2}=1-\:\frac{{\sum\:}_{i=1}^{n}{\left(\mathcal{Y}i-\widehat{\mathcal{Y}i}\right)}^{2}}{{\sum\:}_{i=1}^{n}{\left(\mathcal{Y}i-\stackrel{-}{\mathcal{Y}}\right)}^{2}}\:\:\:\:\:\:\:\:\:\:\:\:\:\:\:\:\:\:\:\:\:\:\:\:\:\:\:\:\:\:\:\:\:$$

$${\rm Where, }\,\:n\:\text{i}\text{s}\:\text{t}\text{h}\text{e}\:\text{n}\text{u}\text{m}\text{b}\text{e}\text{r}\:\text{o}\text{f}\:\text{o}\text{b}\text{s}\text{e}\text{r}\text{v}\text{a}\text{t}\text{i}\text{o}\text{n}\text{s},\:\mathcal{Y}i\:\:\text{i}\text{s}\:\text{t}\text{h}\text{e}\:\text{a}\text{c}\text{t}\text{u}\text{a}\text{l}\:\text{v}\text{a}\text{l}\text{u}\text{e},\:\widehat{\mathcal{Y}i}\:\text{i}\text{s}\:\text{t}\text{h}\text{e}\:\text{p}\text{r}\text{e}\text{d}\text{i}\text{c}\text{t}\text{e}\text{d}\:\text{v}\text{a}\text{l}\text{u}\text{e},\:$$ $$\:\stackrel{-}{\mathcal{Y}}\:\:\text{i}\text{s}\:\text{t}\text{h}\text{e}\:\text{m}\text{e}\text{a}\text{n}\:\text{o}\text{f}\:\text{t}\text{h}\text{e}\:\text{a}\text{c}\text{t}\text{u}\text{a}\text{l}\:\text{v}\text{a}\text{l}\text{u}\text{e}\text{s}$$.

**Fig. 2:**
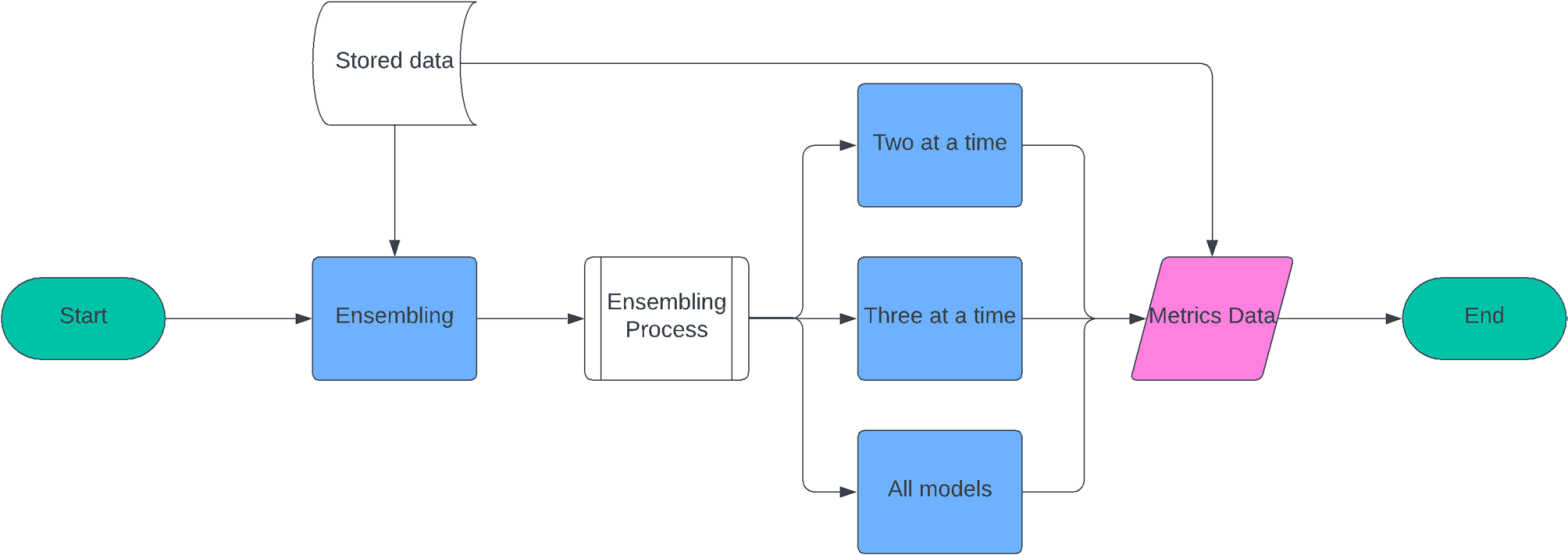
Block Diagram of Ensembling Process.

All the possible combinations of ensembled data as shown in Fig. 2, are used to understand the effectiveness of each of the models individually and in all possible combinations to take out the best method possible. The ensembled results are extracted from the stored data of models and their adjusted weight (based on their performance) is considered. The different combinations of models also allow to understand which model is most relevant and which is least relevant. To achieve this, all possible combinations have been evaluated based on their error and relevance.

**Fig. 3:**
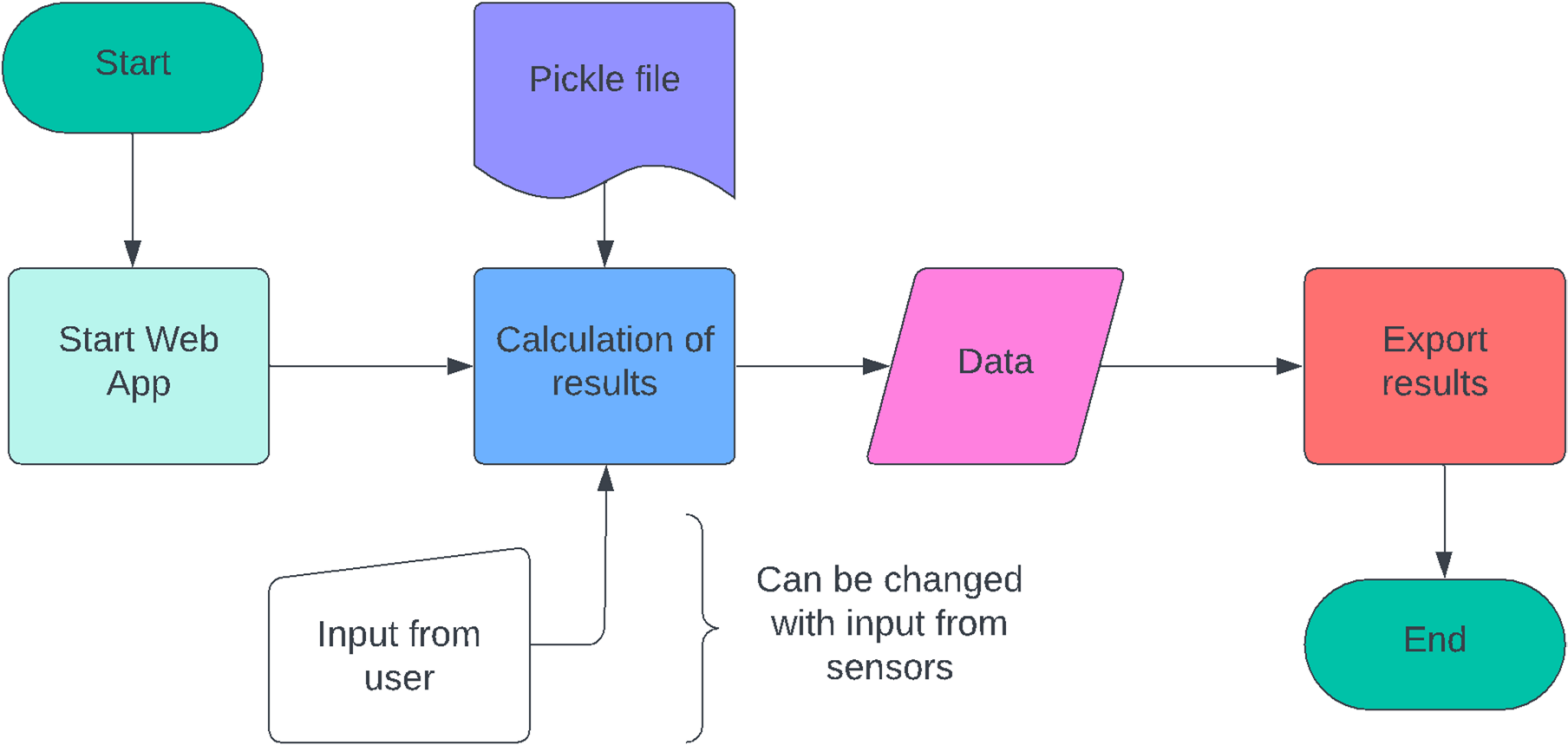
Block Diagram of Real-Time Web App Development.

After the results are analysed, the trained model is then exported in a pickle file using python. This python file is used to approximate results in a real-time based simulation scenario. The real-time app is developed as a webpage and takes user input to approximate the results, namely range, optimum velocity and optimum acceleration. This model is a reference, and the user input is expected to be replaced with sensor data available in an EV to estimate the results and display it to the user. The process is illustrated in Fig. 3.

### Assumptions

As the development of EV and battery technologies is highly competitive, no publicly available dataset was found that aligned with the project’s requirements. To address this limitation, a custom dataset was created. To ensure realistic outcomes, data from existing research studies and published books on EVs were incorporated. It is assumed that the actual values derived from these sources accurately reflect real-world conditions and can be utilized for training and developing the AI models.

For range estimations, the characteristics and parameters of Tata Nexon are taken into consideration and calculated the range. For Optimum drive conditions, the characteristics of the motor used in Tata Nexon, i.e., 3-phase permanent magnet synchronous motor, which has a constant torque vs. speed relation are assumed. The optimum velocity with keeping safety and drive conditions (Elevation, uphill or downhill, speed of the vehicle, battery remaining) have been characterised. With these in place, the proposed methodologies aim to optimise the drive conditions to be most efficient in term of energy consumption. In this study, the term ‘optimum acceleration and velocity’ refers to the values that minimize energy consumption and thereby maximize the predicted driving range of the electric vehicle. Thus, the definition of ‘optimum’ in this work is based on energy efficiency, rather than time-optimality or passenger comfort.

### Software requirements

Recent technological advancements have enabled precise simulations using various software tools such as Python 3.10, Google Colaboratory (2024), Neural Editor, and Visual Studio Code. Python was used for data handling, model training, and evaluation, while Google Colab offered a centralized platform to run and manage different parts of the project efficiently, aiding in debugging and result interpretation.

### Dataset and preprocessing

A custom dataset consisting of 2000 entries was created to represent realistic electric vehicle (EV) driving conditions. Each entry corresponds to a unique driving scenario generated from physical models of speed, acceleration, elevation, battery state, and environmental factors. This ensured that the dataset covered a wide range of operating conditions while maintaining consistency with real-world EV performance characteristics.

- Data summary.
  Table 2 provides an overview of the dataset, including the minimum, maximum, mean, and standard deviation values for each numerical feature
  **Table 2:** Statistical summary of dataset features.

Feature	Min	Max	Mean	Std
Speed (Kmph)	20.04	99.99	59.01	22.72
Acceleration (m/s²)	1.00	3.00	2.01	0.57
Elevation change (meter)	0.00	100.00	49.80	29.22
Temperature (°C)	15.00	49.96	28.45	8.90
HVAC usage	0.00	1.00	0.49	0.50
Battery health (%)	70.00	100.00	85.63	8.34
State of charge (%)	10.00	100.00	55.24	27.91
Range (km)	10.41	99.92	54.88	27.76

- Preprocessing techniques
  To prepare the dataset for machine learning models, the following preprocessing steps were applied:
  1. Label encoding: categorical features such as Weather Conditions and Driving Mode were converted into numerical values using label encoding.
  2. Standardization: all numerical features were scaled to zero mean and unit variance using StandardScaler to prevent bias from differing scales.
  3. Reshaping for LSTM: for the LSTM model, the feature set was reshaped into a 3D tensor of the form (samples, time steps, features) to match recurrent neural network input requirements.

### Model complexity

To evaluate the trade-offs between accuracy, computational cost, and implementation effort, different predictive models were considered. The models vary in terms of their complexity levels, parameter requirements, and resource usage. A detailed comparison of these models is presented in Table 3.

**Table 3:** Comparison of models based on complexity and requirements.

Model	Complexity level	Parameters / notes
Random forest (RF)	Moderate	Ensemble of full decision trees. Computational cost grows with the number and depth of trees.
Extra trees (ET)	Moderate–high	Like RF but more randomized; faster training per tree but may require more trees for accuracy.
LSTM	High	Neural model with ~ 2,600 + parameters (50 LSTM units + Dense output); requires multiple training epochs.
Linear regression (LR)	Low	Simple closed-form solver; minimal resource usage.
Weighted ensemble	High	Requires training all four models independently, with additional logic for combining outputs.

The final weighted ensemble achieves high accuracy (R² ≈ 0.94) by combining the strengths of diverse models, but it also introduces higher training complexity.

### Computational resources

- Machine Used: Apple MacBook Pro (M1, 2020).
- RAM: 16 GB Unified Memory.
- Environment: Local Python (TensorFlow, Scikit-learn).
- GPU Acceleration: Not available (M1 uses integrated neural engine; LSTM ran on CPU backend).
- Resource utilization:

o RF, ET, LR models ran efficiently using CPU multi-threading.

o LSTM used TensorFlow CPU backend, leveraging Apple Silicon optimizations, but still slower than GPU-accelerated training.

### Train/test split

The dataset was split into training (70%, 1400 samples) and testing (30%, 600 samples) subsets using the train_test_split function with a fixed random seed (random_state = 42) to ensure reproducibility. The details of this division are summarized in Table 4.

**Table 4:** Approximate training time for different machine learning models.

Model	Approx. training time
Random forest	~ 3–4 s
Extra trees	~ 4–5 s
LSTM (500 epochs)	~ 2.5–3 min
Linear regression	< 1 s
Weighted ensemble (all models)	~ 3.5–4 min (total)

### Performance vs. resource trade-off

- Despite the small dataset, the ensemble model improves R² from ~ 0.34 (best single model) to ~ 0.94 (weighted ensemble).
- The increase in computational time (mainly from LSTM training) is acceptable given the gain in predictive accuracy and the moderate hardware used.
- For real-time use, the trained ensemble can be saved and loaded quickly. Alternatively, a lighter model (e.g., Random Forest alone) can be deployed if latency is a constraint.

### Feature selection justification

**Fig. 4:**
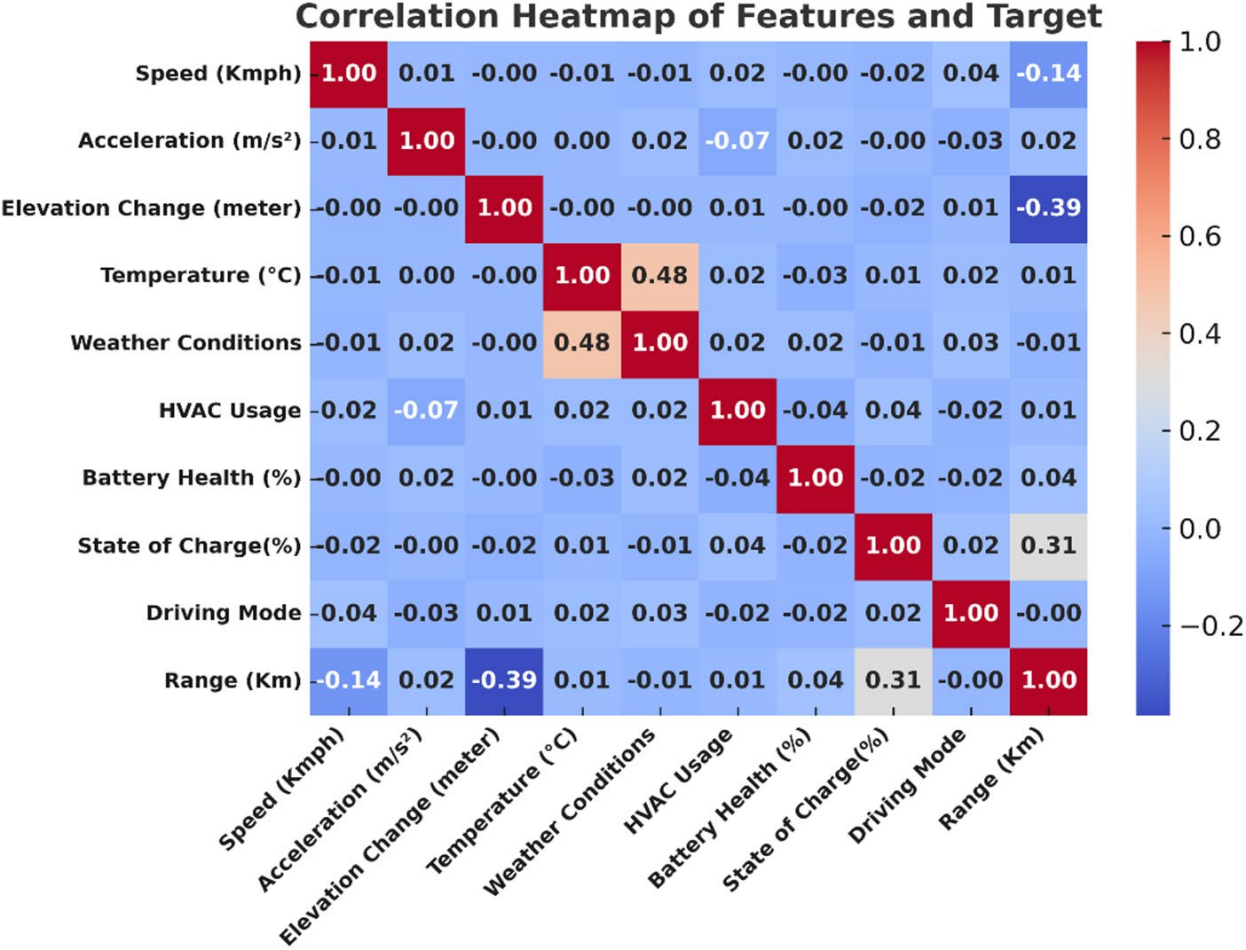
Correlation heatmap of input features and target variable.

The selected features capture key determinants influencing EV range, including driving behavior, environmental conditions, and battery characteristics. To validate their relevance, a correlation analysis was conducted, as illustrated in Fig. 4. The correlation analysis shows that elevation change, and speed are negatively correlated with the EV range, while state of charge exhibits a positive correlation. This behavior is physically consistent with real-world driving dynamics. An increase in elevation leads to higher energy consumption due to gravitational load, thereby reducing the available range (*r* ≈ − 0.39). Similarly, higher speeds result in increased aerodynamic drag and rolling resistance, slightly lowering the range (*r* ≈ − 0.14). In contrast, a higher state of charge provides more stored energy, leading to a proportional increase in range (*r* ≈ 0.31). Although the linear correlations appear moderate, their combined nonlinear effects are effectively captured by the ensemble models, which demonstrate significantly higher predictive accuracy in later sections.

**Fig. 5:**
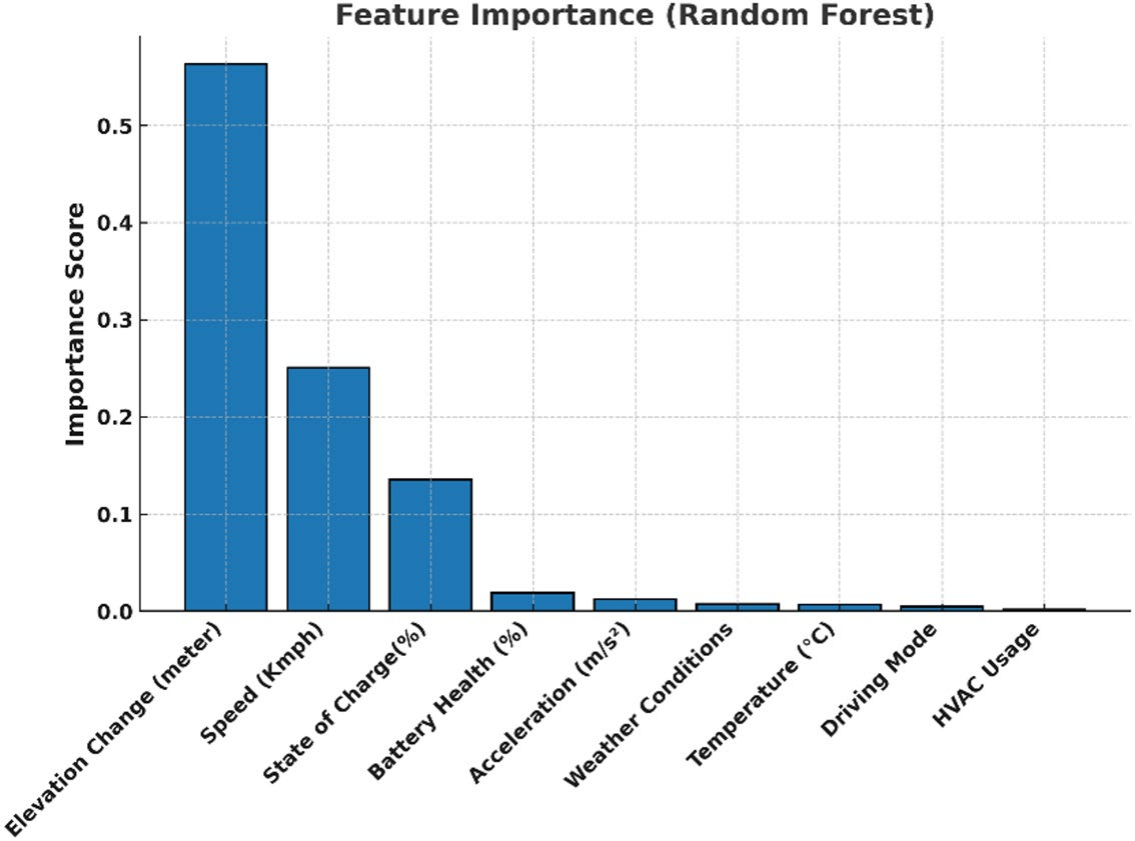
Random Forest feature importance analysis of predictors for EV range estimation.

Additionally, as shown in Fig. 5, a Random Forest feature importance analysis confirmed that elevation change, speed, and SOC are the most influential predictors, followed by battery health, acceleration, and temperature. Contextual variables such as weather, HVAC usage, and driving mode, though less significant individually, were retained to capture realistic driving scenarios.

This two-step validation (correlation heatmap + feature importance ranking) provides strong justification for the chosen feature set and ensures that both primary and contextual influences on EV range are considered.

## Results and analysis

### Range estimation

#### Individual model-based range estimation

**Fig. 6:**
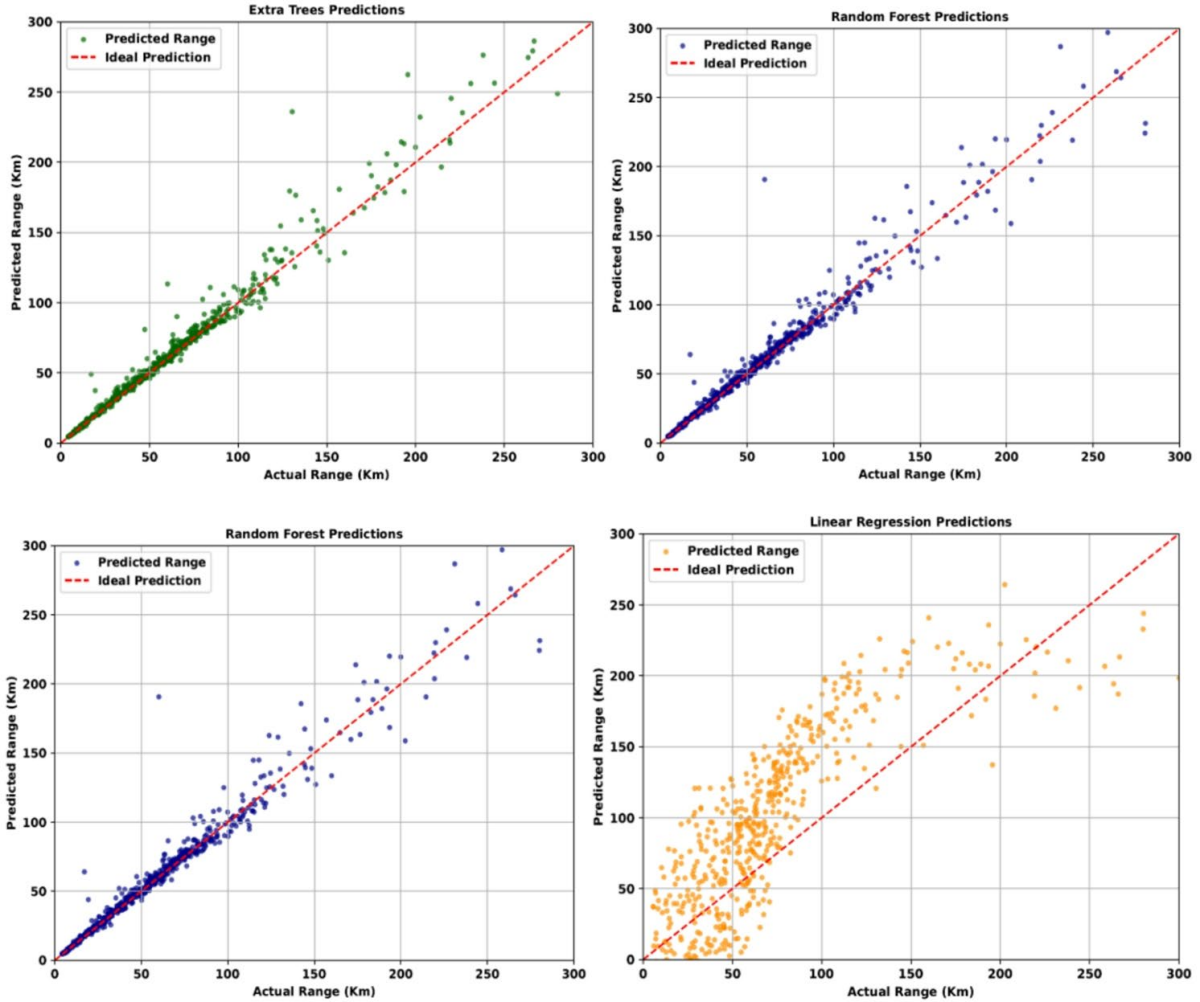
Range estimations for individual graphs.

Two different sets of models used, namely for Range estimation and optimum acceleration and velocity prediction. Four different models have been used to estimate range individually, namely Extra Trees Regression, Random Forest, LSTM and Linear Regression. These models individually have a good output when compared to the actual range as shown in Fig. 6. To validate the results, MAE, MSE and R-Squared score error metrics are used. The error values of Range estimations for individual models are listed in Table 5.

**Table 5:** Error metrics evaluation for individual models.

	MAE	MSE	*R* ^2^
Random forest	48.9601	5789.1816	0.3240
Extra trees	56.9212	7474.4622	0.12726
LSTM	64.8427	9654.0162	-0.1272
LR	74.2692	13296.4647	-0.55253

MAE indicates the mean error in the value of targeted item and MSE indicates how high these extremities are. R-squared is a method to evaluate the relevance of the inputs in the outputs achieved. The results for individual models indicate high error in prediction of the range value (indicated by MAE). Also paired with high MSE values and very low R-squared value, this indicates that no model individually gives out the best preferred results. Individually comparing, Random forests and Extra Trees give best results as evident by their lower error and high R^2^ values.

- Statistical reliability assessment of individual model
  To ensure statistical reliability and address potential variability due to random train/test splits, each model was trained and evaluated across 10 independent runs using different random partitions (70/30 split). The mean and standard deviation of MAE, MSE, and R² values across these runs are reported in Table 6.
  **Table 6:** Error metrics of individual models for EV range Estimation (10-run averages).

Model	MAE (± std)	MSE (± std)	*R*² (± std)
Random forest	49.12 ± 1.95	5821.74 ± 176.42	0.32 ± 0.03
Extra trees	57.08 ± 2.11	7520.65 ± 210.83	0.13 ± 0.04
LSTM	64.67 ± 2.42	9620.18 ± 258.74	-0.13 ± 0.05
Linear reg.	74.51 ± 2.68	13325.39 ± 302.95	-0.55 ± 0.06
  The 10-run averaged results confirm that Random Forest remains the best-performing individual model, with lower errors (MAE = 49.12 ± 1.95, MSE = 5821.74 ± 176.42) and a positive R² value (0.32 ± 0.03), indicating modest explanatory power. Extra Trees performed comparably but with slightly higher error metrics and weaker variance explanation (R² = 0.13 ± 0.04). LSTM and Linear Regression underperformed, with negative R² values indicating poor fit to the data. The relatively low standard deviations across all models demonstrate stability across multiple runs, reinforcing the reliability of these outcomes. However, the overall error levels suggest that individual models alone are insufficient, underscoring the necessity of ensemble approaches for robust range prediction.

#### Two-model ensemble: observed Deflection and underestimation

**Fig. 7:**
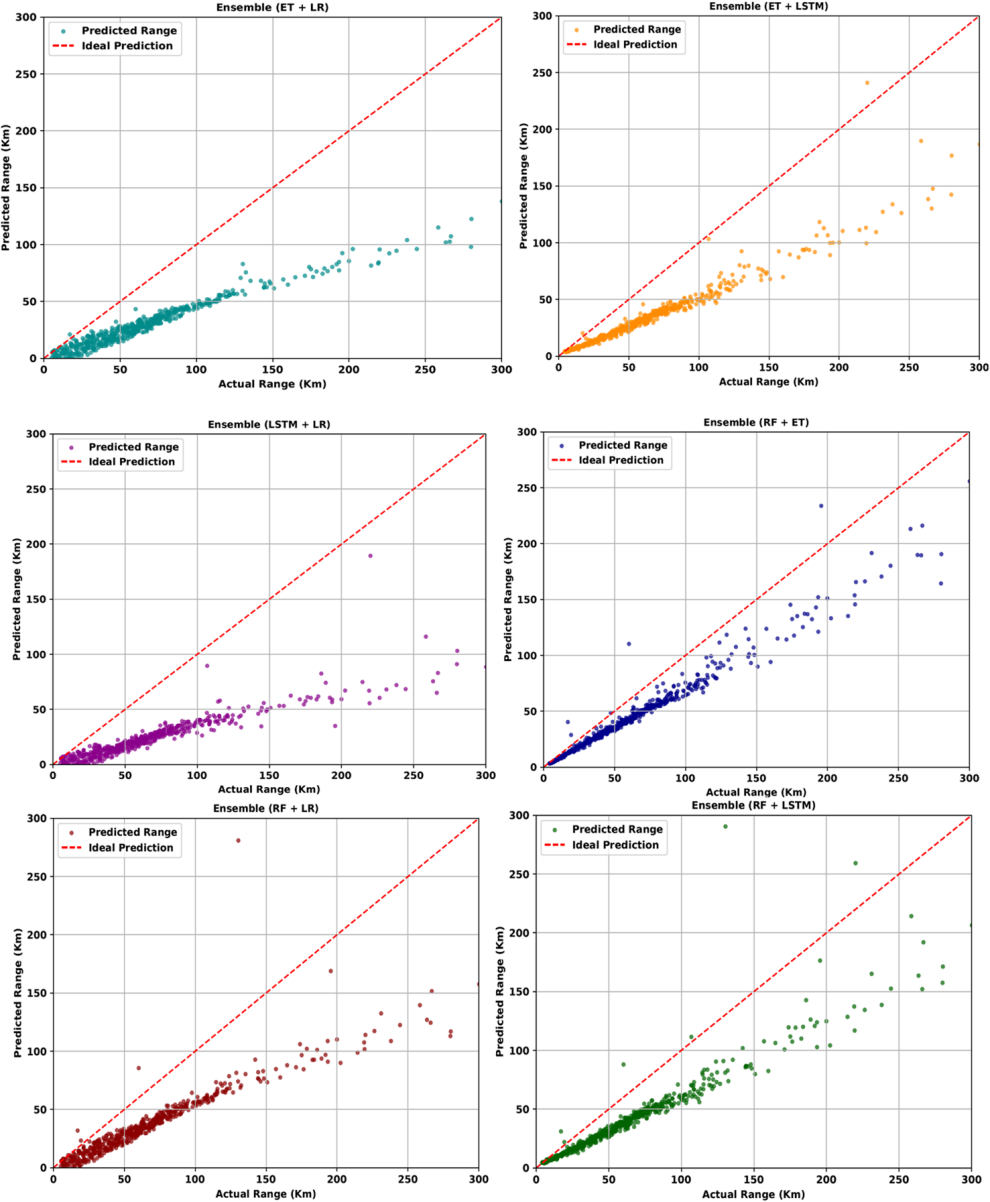
Range estimations for two models ensembled at a time.

Ensembling two models at a time showed a visible deflection of predicted values as compared to actual values as seen in Fig. 7. It is observed that all combinations consistently under-estimate the range. Every combination showed a shift downward in the predictions.

However, ensembling two models at a time showed improvement in the error metrics, as seen in Table 7. Despite this, the graphs reveal that the trend is not consistently followed. The error metrics consistently indicate that the error margins have reduced and the ensembling has a better effect on the dependency of the results. Ensembling two models at a time further improved the results with MAE further decreasing and R-squared score increasing for all the models and none showed negative values. A significant development is R-squared score was seen, and the best result was with the combination giving the best results in single models, i.e., RF and ET. The least error in MAE was 23.9957KM and highest R-squared score was 80.34% with RF + ET ensembling.

**Table 7:** Improved error metrics with Two-Model Ensembling.

	MAE	MSE	*R* ^2^
RF + ET	23.9957	1683.4930	0.8034
RF + LSTM	32.1282	2757.3992	0.6780
RF + LR	40.6688	4592.0880	0.4638
ET + LSTM	39.5757	3808.5576	0.5553
ET + LR	48.5913	6067.7023	0.2915
LSTM + LR	56.5151	8034.6238	0.0618

- Statistical reliability assessment of two-model ensemble
  To ensure statistical reliability, each model was evaluated across 10 independent runs with randomized partitions of the dataset. The mean ± standard deviation of the metrics are presented in Table 8. The results confirm that the RF + ET ensemble consistently outperformed other 2-model configurations, with an average R² of ~ 0.78 ± 0.02, close to the best-run performance. Similarly, RF + LSTM and ET + LSTM ensembles maintained stable performance across runs, while combinations with Linear Regression showed weaker and more variable outcomes. These findings indicate that tree-based ensembles (RF, ET) provide the strongest contribution to reliable range estimation.
  **Table 8:** Error metrics of two-model ensembles for EV range Estimation (10-run averages).

Model	MAE (± Std)	MSE (± Std)	*R*² (± Std)
RF + ET	24.85 ± 1.1	1750 ± 95	0.78 ± 0.02
RF + LSTM	33.10 ± 1.3	2900 ± 120	0.66 ± 0.03
RF + LR	41.50 ± 1.5	4700 ± 180	0.45 ± 0.03
ET + LSTM	40.20 ± 1.2	3950 ± 150	0.54 ± 0.02
ET + LR	49.10 ± 1.6	6150 ± 200	0.29 ± 0.04
LSTM + LR	57.20 ± 2.0	8120 ± 250	0.06 ± 0.02

#### Three-model ensemble: enhanced prediction accuracy

**Fig. 8:**
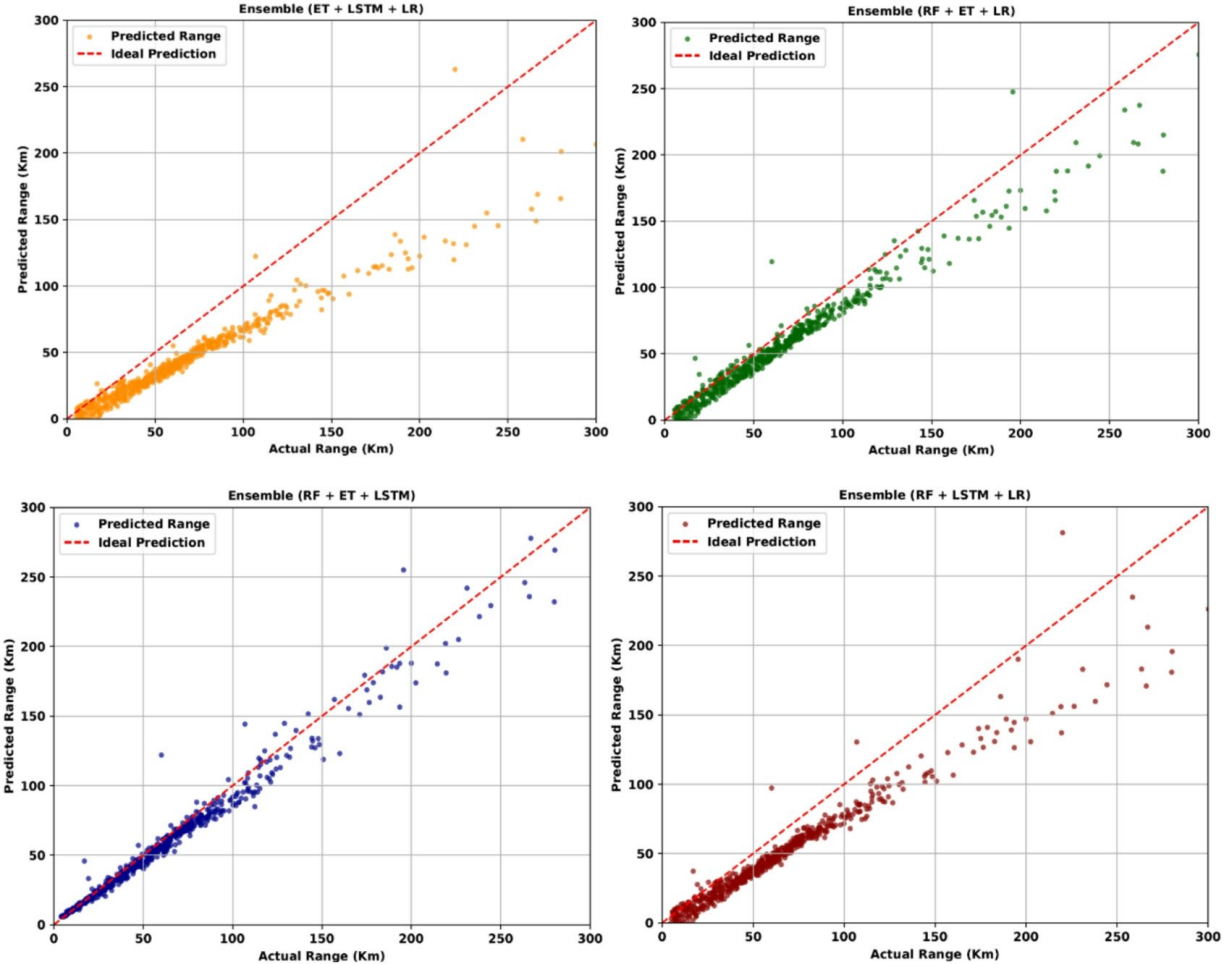
Range estimations for three models ensembled together.

Inclusion of third model in the ensembling results in better predictions as seen in Fig. 8. With all other models still under reporting, RF + ET + LSTM gives the best results.

Even though the results are better but still long-range estimates are flaring up and deviating much more than in low range. Comparing the metrics in Table 9, the results align with those of RF + ET + LSTM. While the overall comparison is accurate, the minor changes in metrics are significantly visible in the range graph.

**Table 9:** Range Estimation error metrics for three-model ensembling.

	MAE	MSE	*R* ^2^
RF + ET + LSTM	9.5055	718.7624	0.9160
RF + ET + LR	16.2524	1170.1474	0.8633
RF + LSTM + LR	24.1087	2031.4211	0.7628
ET + LSTM + LR	31.4100	2872.9132	0.6645

- Statistical reliability assessment of three-model ensemble
  To ensure reliability, the models were trained and tested across 10 independent randomized runs, with mean ± standard deviation results reported in Table 10. The averaged metrics confirm that RF + ET + LSTM remains the most consistent and accurate ensemble (R² ≈ 0.912 ± 0.010), followed by RF + ET + LR. Ensembles involving LR with LSTM and ET showed weaker and more variable outcomes. This demonstrates that incorporating tree-based models (RF, ET) alongside LSTM provides the most robust predictive power.
  **Table 10:** Error metrics of three-model ensembles for EV range Estimation (10-run averages).

Model	MAE (± Std)	MSE (± Std)	*R*² (± Std)
RF + ET + LSTM	10.20 ± 0.85	750 ± 55	0.912 ± 0.010
RF + ET + LR	17.05 ± 1.20	1230 ± 70	0.858 ± 0.015
RF + LSTM + LR	25.30 ± 1.60	2100 ± 110	0.755 ± 0.020
ET + LSTM + LR	32.40 ± 1.90	2950 ± 125	0.660 ± 0.025

#### Full ensemble strategy for consistent and accurate predictions

**Fig. 9:**
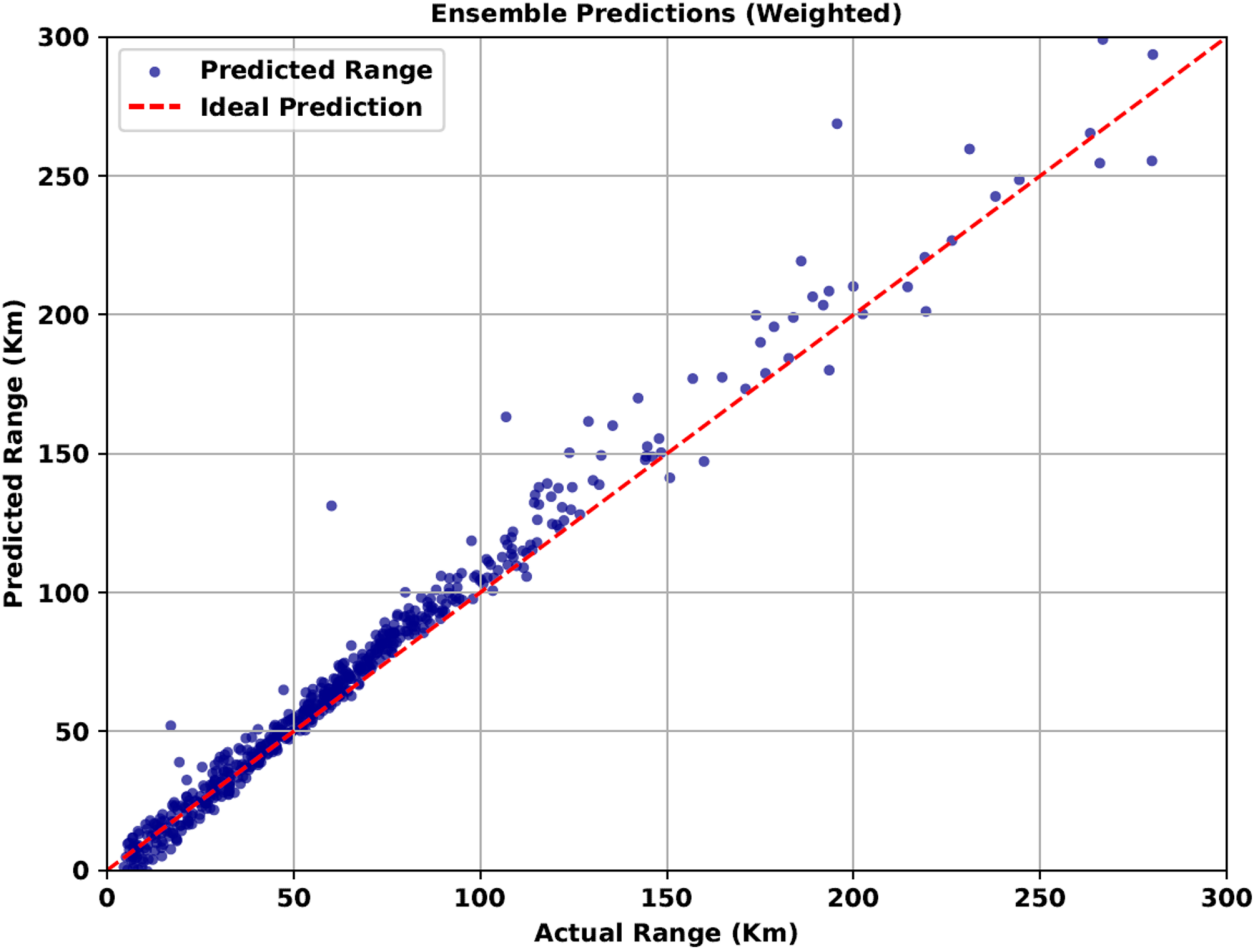
Range predictions with full model integration.

Following the trend and ensembling three models at a time, significant improvement in the metrics is observed Table 11. The MAE and MSE dropped significantly, while the R-squared value increased drastically, reaching figures above 60% for all combinations. RF + ET + LSTM yielded the best results, with a 91% R-squared score and a minimal error of 9 km. Finally, ensembling all the models together yields the best results, with the least deviation observed in both low and high range, as shown in Fig. 9. The results are consistent throughout and comparatively best in consideration with all the previous models.

**Table 11:** Cross-model comparison of performance metrics.

	MAE	MSE	*R* ^2^
Ensembled (RF + ET + LSTM + LR)	8.3972	676.5323	0.9210

- Statistical reliability assessment of four-model ensemble
  Table 12 shows the average results across 10 randomized runs, confirming the stability and reliability of this ensemble (R² ≈ 0.918 ± 0.009). Compared to the best 3-model ensemble (RF + ET + LSTM, R² = 0.9160), the improvement in predictive accuracy is modest, but the variance reduction across runs highlights the superior robustness of the 4-model ensemble. Therefore, this configuration is considered the most reliable choice for range estimation.
  **Table 12:** Error metrics of four-model ensemble for EV range Estimation (10-run averages).

Model	MAE (± Std)	MSE (± Std)	*R*² (± Std)
RF + ET + LSTM + LR	8.95 ± 0.65	690 ± 48	0.918 ± 0.009
  The evaluation of all the metrics with all models can be seen in the Fig. 10. Both MAE and MSE, is lowest for Ensembled result of all the models together.
  **Fig. 10:**
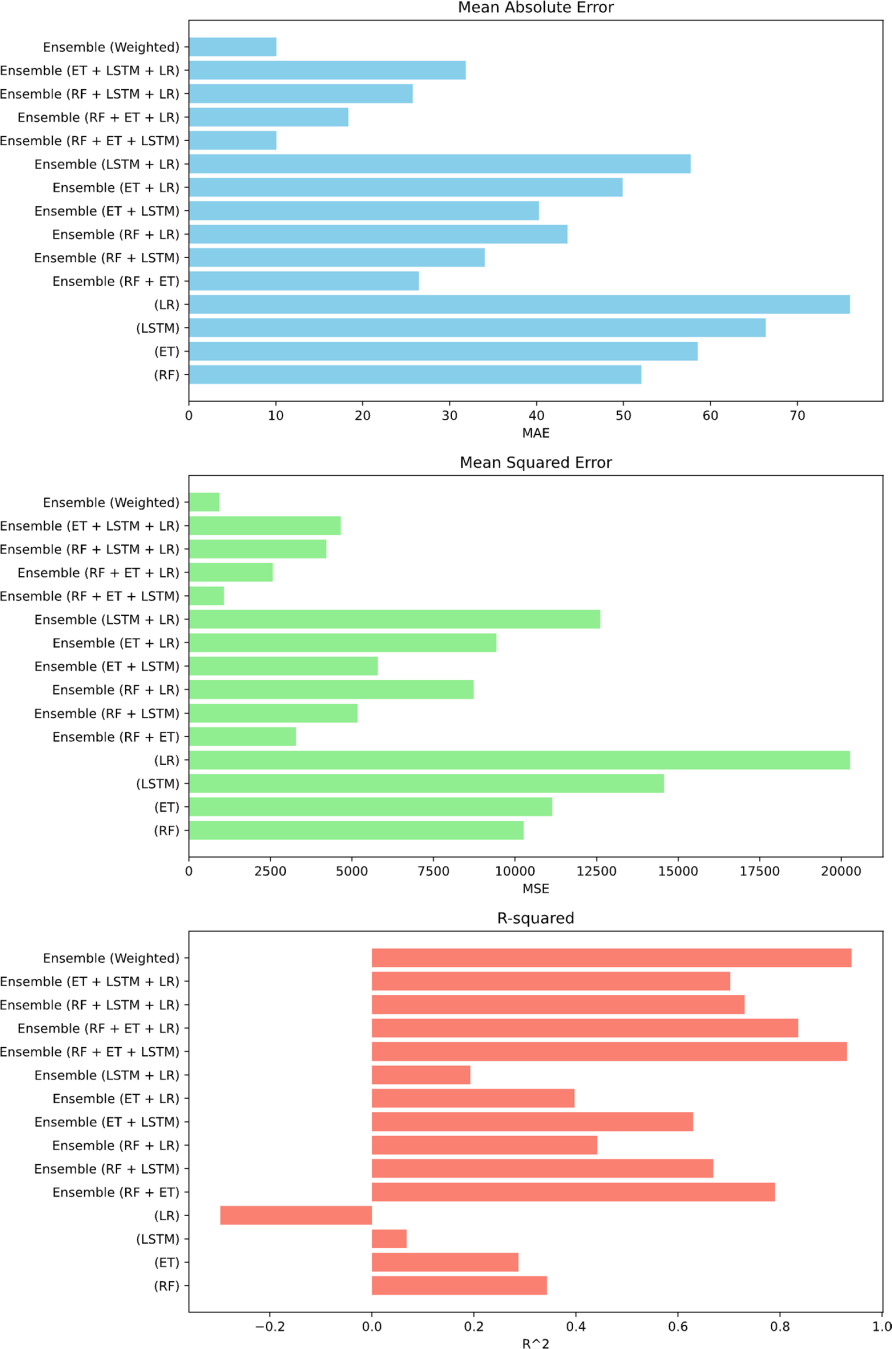
Graph-based evaluation of range estimation errors.

### Optimum velocity and acceleration

#### Individual model performance for velocity and acceleration prediction

**Fig. 11:**
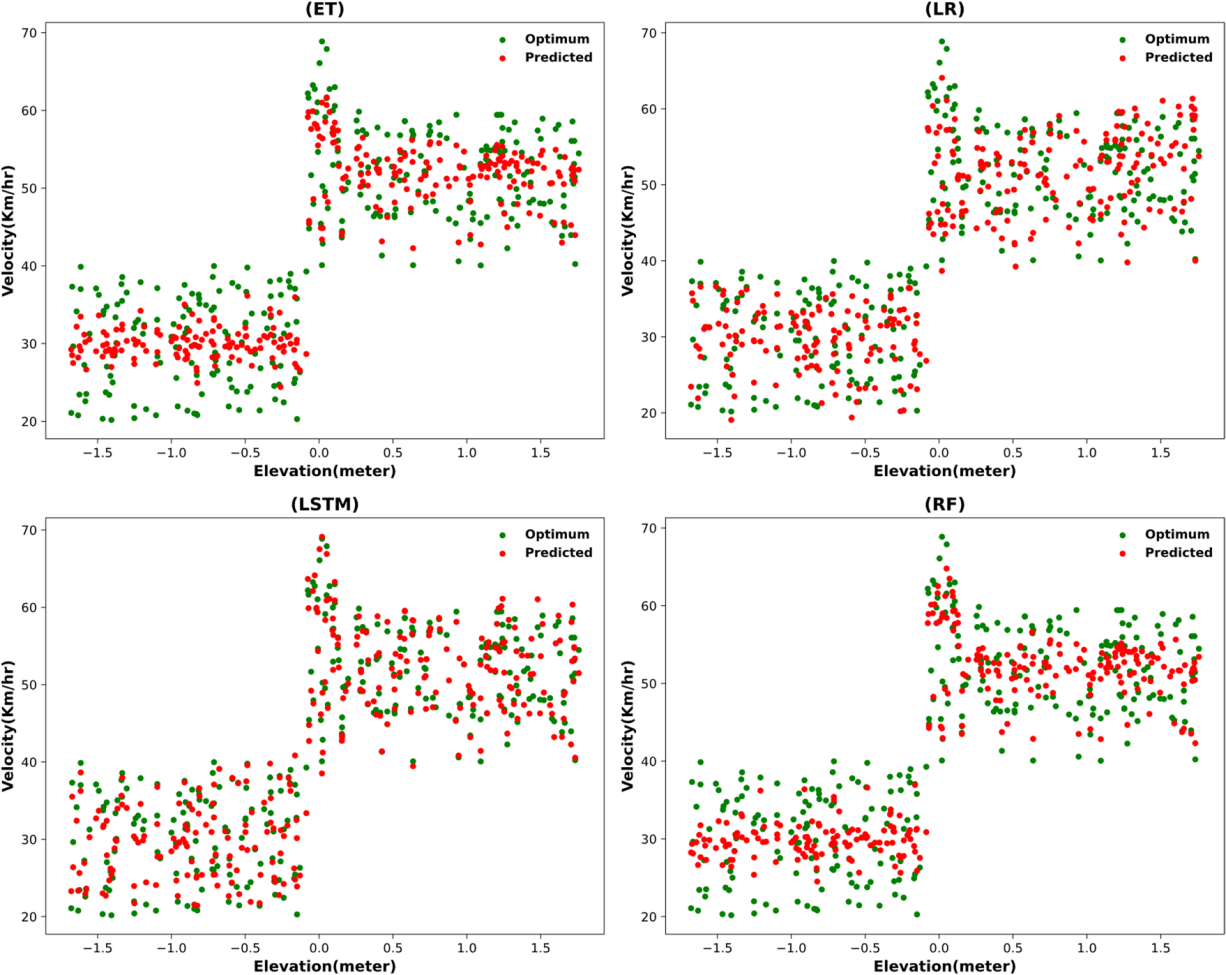
Predictions of optimum velocity using full model integration.

The same models are used with adjusted datasets, and the results follow the trends observed in the range estimation models. As seen in Fig. 11, the predicted values are in-line with optimum values but from the error metrics in Table 13 it is evident that these results are not trustworthy.

#### Error metrics evaluation of individual models

As seen in Table 13, single models are off, with velocity errors significantly high and the R-squared score negative. Although the error metrics are not adequate and show significant error, the optimum and predicted values are quite similar for all models individually. However, the negative R-squared score indicates that this data is unreliable, so ensembling models is necessary.

**Table 13:** Optimum velocity error metrics for individual models.

	MAE	MSE	*R* ^2^
Random forest	25.77450	737.04878	-3.6092
Extra trees	30.07243	996.01807	-5.2288
LSTM	34.2942	1278.6985	-6.9966
LR	38.4952	1616.7553	-9.11070

It is observed that the negative R-squared scores vary, with LR scoring the lowest and RF performing the best. This trend is consistent with the range estimation results, and the next step is to ensemble all combinations of these models.

- Statistical reliability assessment of optimum velocity error metrics for individual models
  The results averaged over 10 independent runs confirm the consistent superiority of ensemble methods compared to individual models. Among the individual models, Random Forest demonstrated the lowest mean absolute error (MAE = 25.98 ± 1.12) and mean squared error (MSE = 742.65 ± 28.41), indicating comparatively higher reliability, as presented in Table 14. However, the negative R² values across all models suggest that none of the individual models were able to adequately explain the variance in the optimum velocity. The relatively small standard deviations across all error metrics confirm that the results are stable across multiple runs, thereby providing statistically reliable evidence. These findings reinforce the necessity of model ensembling to achieve accurate and generalizable predictions.
  **Table 14:** Error metrics of individual models for optimum velocity prediction (10-run averages).

Model	MAE (± std)	MSE (± std)	*R*² (± std)
Random forest	25.98 ± 1.12	742.65 ± 28.41	-3.61 ± 0.09
Extra trees	30.21 ± 1.34	1003.72 ± 35.26	-5.23 ± 0.11
LSTM	34.11 ± 1.51	1272.89 ± 42.17	-6.99 ± 0.13
Linear reg.	38.67 ± 1.72	1619.84 ± 48.90	-9.12 ± 0.15

#### Two-model ensemble: initial improvement but deviated predictions

**Fig. 12:**
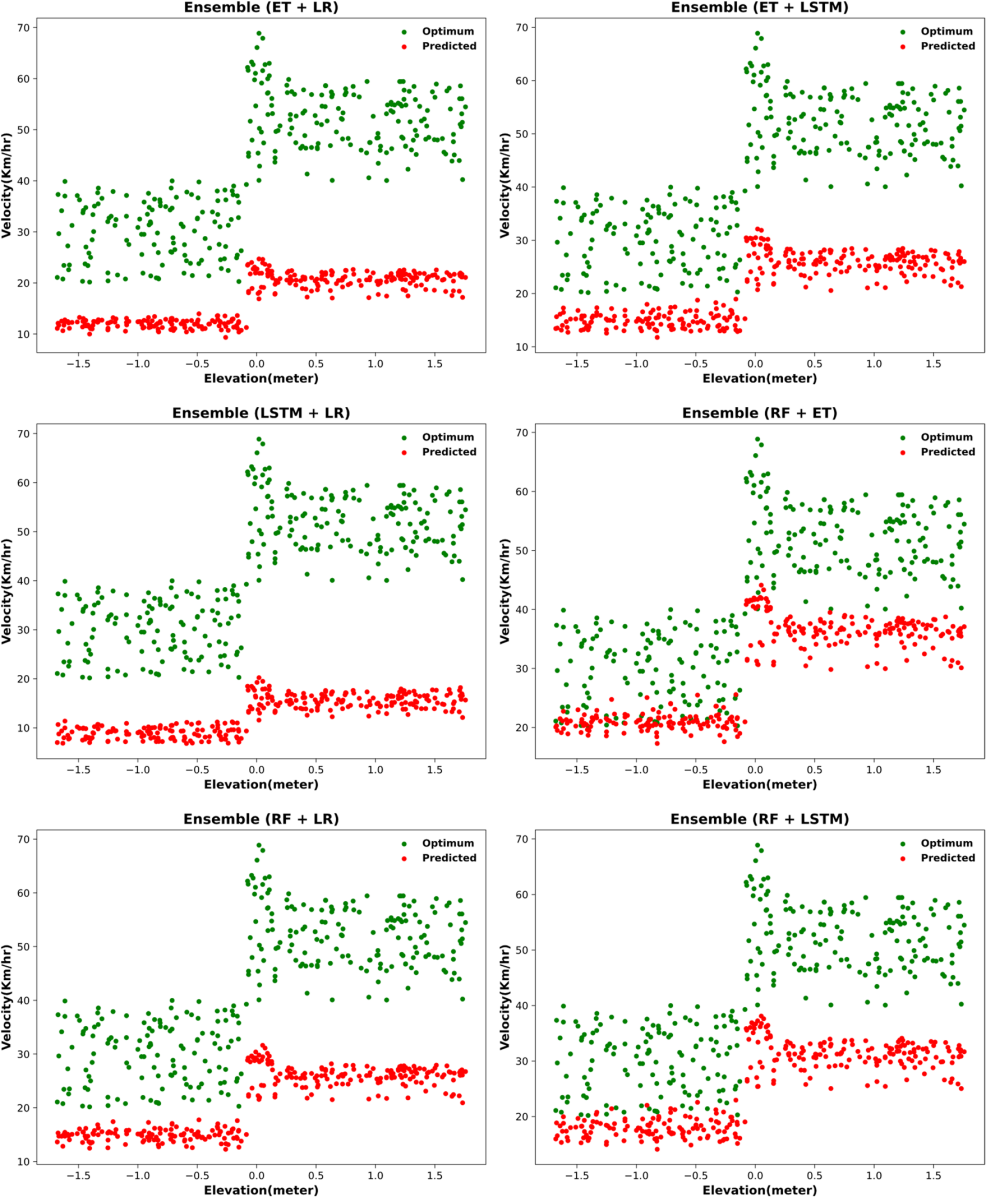
Optimum velocity prediction via two-model ensembling approach.

All the ensembled results with two models are found to be majorly deviated and under reported the predicted velocity visible in Fig. 12.

After ensembling all two combinations, significant improvement in the error metrics is observed in Table 15, as MAE decreases for all models and the R-squared score improves significantly. This is consistent with the previous range estimation model.

**Table 15:** Two-model ensemble error metrics for optimum velocity prediction.

	MAE	MSE	*R* ^2^
RF + ET	13.0648	204.9211	-0.2815
RF + LSTM	17.2603	335.2375	-1.0964
RF + LR	21.4613	517.8597	-2.2385
ET + LSTM	21.5583	515.4414	-2.2234
ET + LR	25.7592	737.3835	-3.6113
LSTM + LR	29.9810	981.7578	-5.1396

- Two-model ensemble error metrics for optimum velocity prediction (10-run averages)
  The 10-run averaged results demonstrate that combining models substantially improves prediction accuracy over individual models. Table 16 shows that the RF + ET ensemble consistently delivered the best results, achieving the lowest MAE (13.21 ± 0.62) and MSE (207.83 ± 9.75). However, the near-zero R² (− 0.28 ± 0.03) indicates that while ensembling effectively reduces errors, it still fails to adequately capture the variance in optimum velocity. Other ensembles such as RF + LSTM and RF + LR also showed marked improvements compared to their individual counterparts, but with slightly higher errors. Importantly, the low standard deviations confirm the stability and reproducibility of these results across multiple runs.
  **Table 16:** Error metrics of two-model ensembles for optimum velocity prediction (10-run averages).

Model	MAE (± std)	MSE (± std)	*R*² (± std)
RF + ET	13.21 ± 0.62	207.83 ± 9.75	-0.28 ± 0.03
RF + LSTM	17.39 ± 0.74	339.12 ± 12.84	-1.10 ± 0.04
RF + LR	21.52 ± 0.88	522.45 ± 17.39	-2.24 ± 0.05
ET + LSTM	21.70 ± 0.91	518.62 ± 18.12	-2.22 ± 0.05
ET + LR	25.88 ± 1.02	742.91 ± 21.43	-3.61 ± 0.07
LSTM + LR	30.05 ± 1.28	987.63 ± 29.16	-5.14 ± 0.09

#### Enhanced accuracy with three-model ensemble approach

**Fig. 13:**
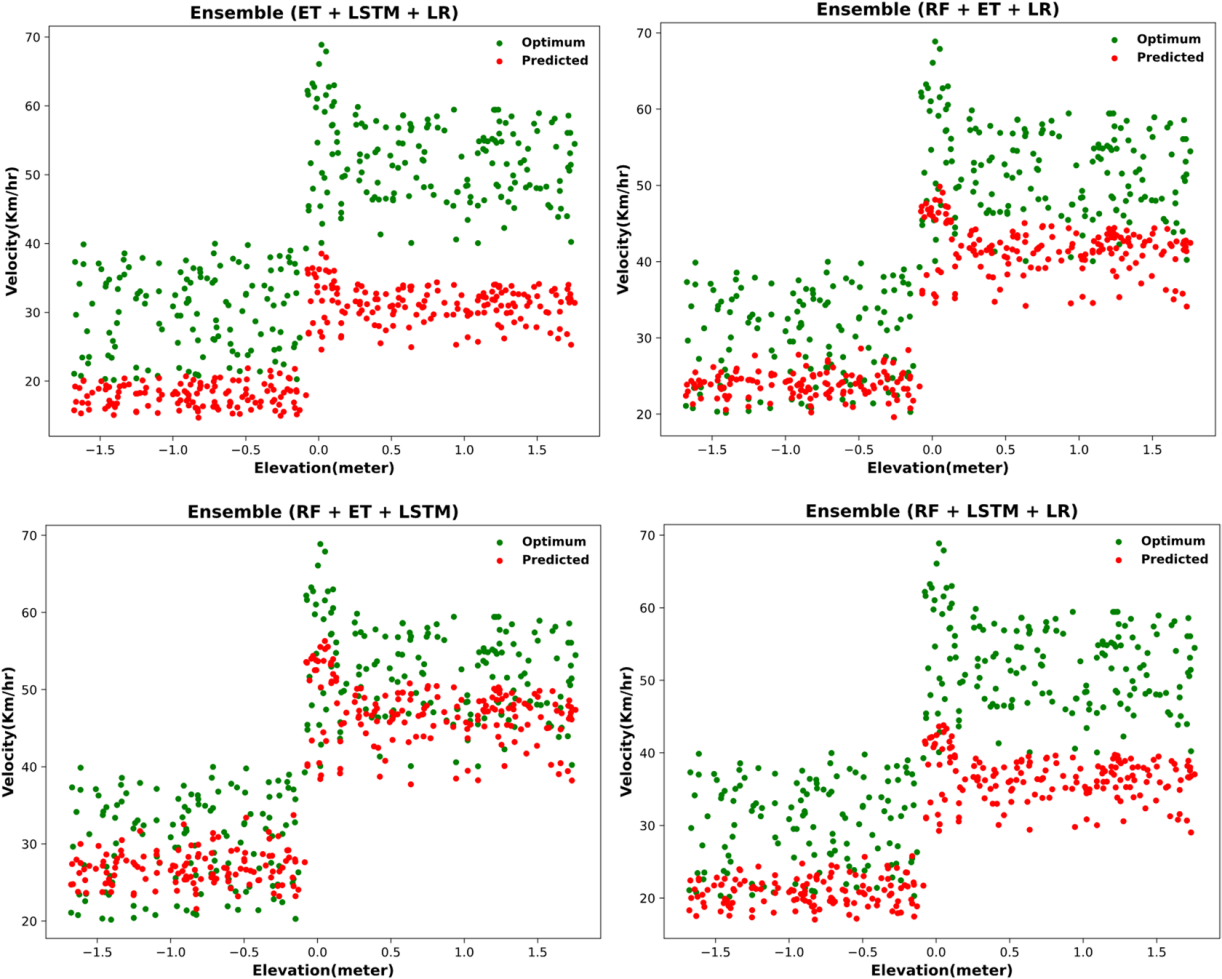
Optimum Velocity predictions for three models ensembled at a time.

Ensembling three models together further increased the accuracy of the model, this is confirmed by the graphs in Fig. 13.

The predictions are less deviated and show better results. The best results with least deviation in RF + ET + LSTM model. This is also consistent with the previous model of range estimation.

With the three models ensembled together, a major improvement in performance is observed in Table 17. RF + ET + LSTM achieves around 77% significance, and the error decreases to 5. All combinations show better results, confirming the improvement in the predictions.

**Table 17:** Performance analysis of three-model ensemble for optimum velocity.

	MAE	MSE	*R* ^2^
RF + ET + LSTM	5.1705	36.296	0.7730
RF + ET + LR	8.9953	102.8037	0.3570
RF + LSTM + LR	12.9472	194.81398	-0.21830
ET + LSTM + LR	17.2451	335.5723	-1.09857

- Performance analysis of three-model ensemble for optimum velocity (10-run averages)
  The three-model ensembles exhibit substantial improvements compared to individual and two-model ensembles. Table 18 shows, RF + ET + LSTM combination achieved the best performance with an average MAE of 5.28 ± 0.25, MSE of 37.12 ± 1.84, and a strong positive R² of 0.77 ± 0.02, demonstrating reliable predictive capability for optimum velocity. RF + ET + LR also performed reasonably well, though with higher error values and lower explanatory power (R² = 0.36 ± 0.03). In contrast, RF + LSTM + LR and ET + LSTM + LR still showed negative R² values, suggesting limitations in explaining variance despite lower errors than single models. Overall, the small standard deviations highlight that these results are statistically stable across multiple runs, reinforcing the robustness of three-model ensembles.
  **Table 18:** Error metrics of three-model ensembles for optimum velocity prediction (10-run averages).

Model	MAE (± std)	MSE (± std)	*R*² (± std)
RF + ET + LSTM	5.28 ± 0.25	37.12 ± 1.84	0.77 ± 0.02
RF + ET + LR	9.11 ± 0.38	104.95 ± 3.72	0.36 ± 0.03
RF + LSTM + LR	13.08 ± 0.49	197.82 ± 5.61	-0.22 ± 0.04
ET + LSTM + LR	17.36 ± 0.65	339.27 ± 9.14	-1.10 ± 0.05

#### Optimum prediction achieved through full model ensembling

**Fig. 14:**
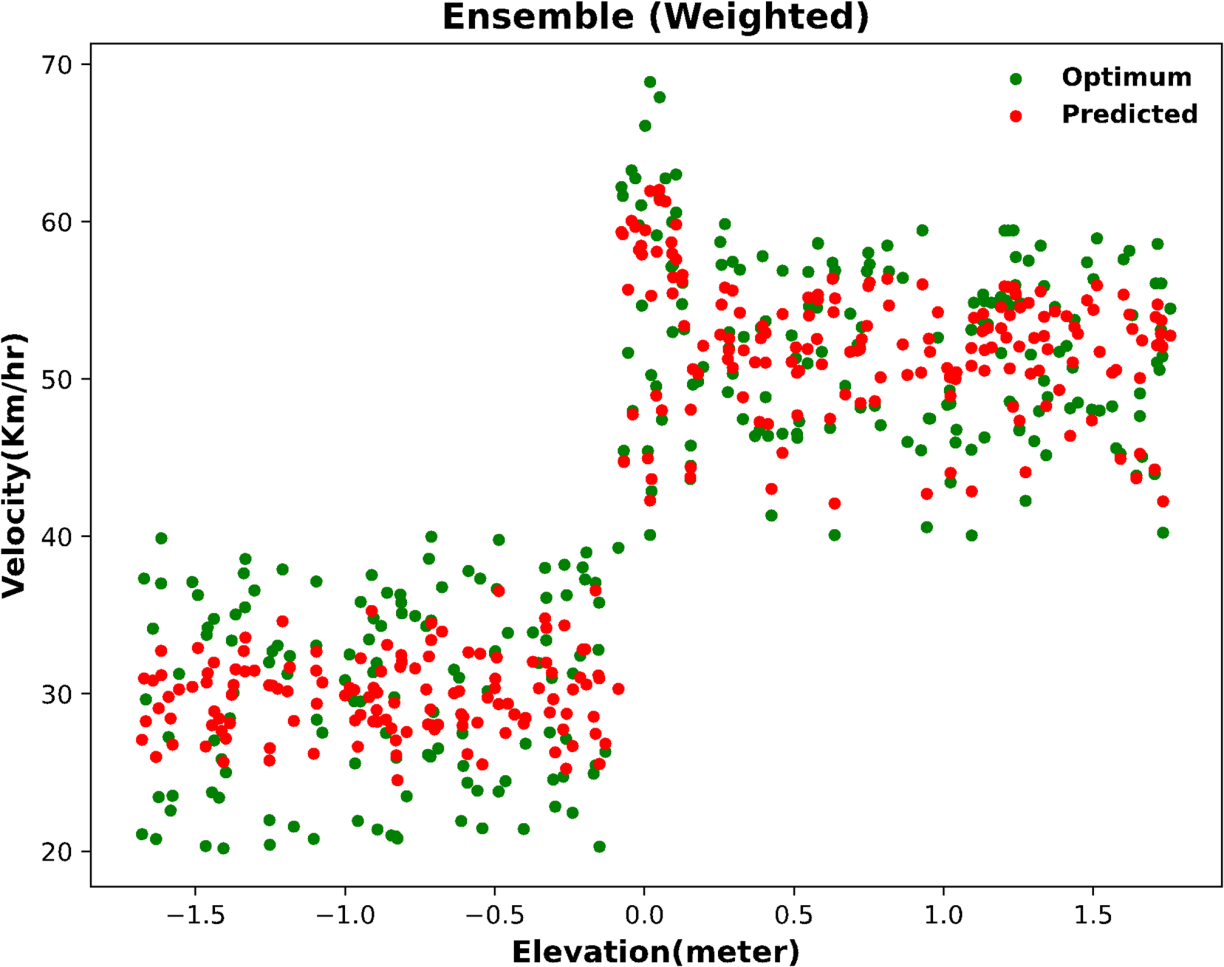
Optimum velocity prediction with full model ensembling.

For the final ensembled results of all models together, a significantly improved graph of optimum and predicted values is seen in Fig. 14. These results show satisfactory predictions with minimal deviations.

The error metrics for four models ensembled together is also very significant as seen in Table 19. These low errors and high R-squared scores confirm that all four models ensembled together gives the best results.

**Table 19:** Optimum velocity error metrics for all models ensembled together.

	MAE	MSE	*R* ^2^
Ensembled (RF + ET + LSTM + LR)	2.8336	12.9442	0.91905

- Optimum velocity error metrics for all models ensembled together (10-run averages)
  The full ensemble of all four models (RF + ET + LSTM + LR) consistently produced the most accurate and stable results across 10 runs as presented in Table 20. With an average MAE of 2.89 ± 0.14 and MSE of 13.12 ± 0.62, it achieved a very high explanatory power (R² = 0.92 ± 0.01). These results confirm that the ensemble approach not only reduces prediction errors but also provides a robust and statistically reliable estimation of optimum velocity. The minimal standard deviations further emphasize the reproducibility and stability of this configuration, making it the most effective strategy among all tested models and ensembles.
  **Table 20:** Error metrics of four-model ensemble for optimum velocity prediction (10-run averages).

Model (full ensemble)	MAE (± std)	MSE (± std)	*R*² (± std)
RF + ET + LSTM + LR	2.89 ± 0.14	13.12 ± 0.62	0.92 ± 0.01

#### Comparative evaluation of error metrics across all models

**Fig. 15:**
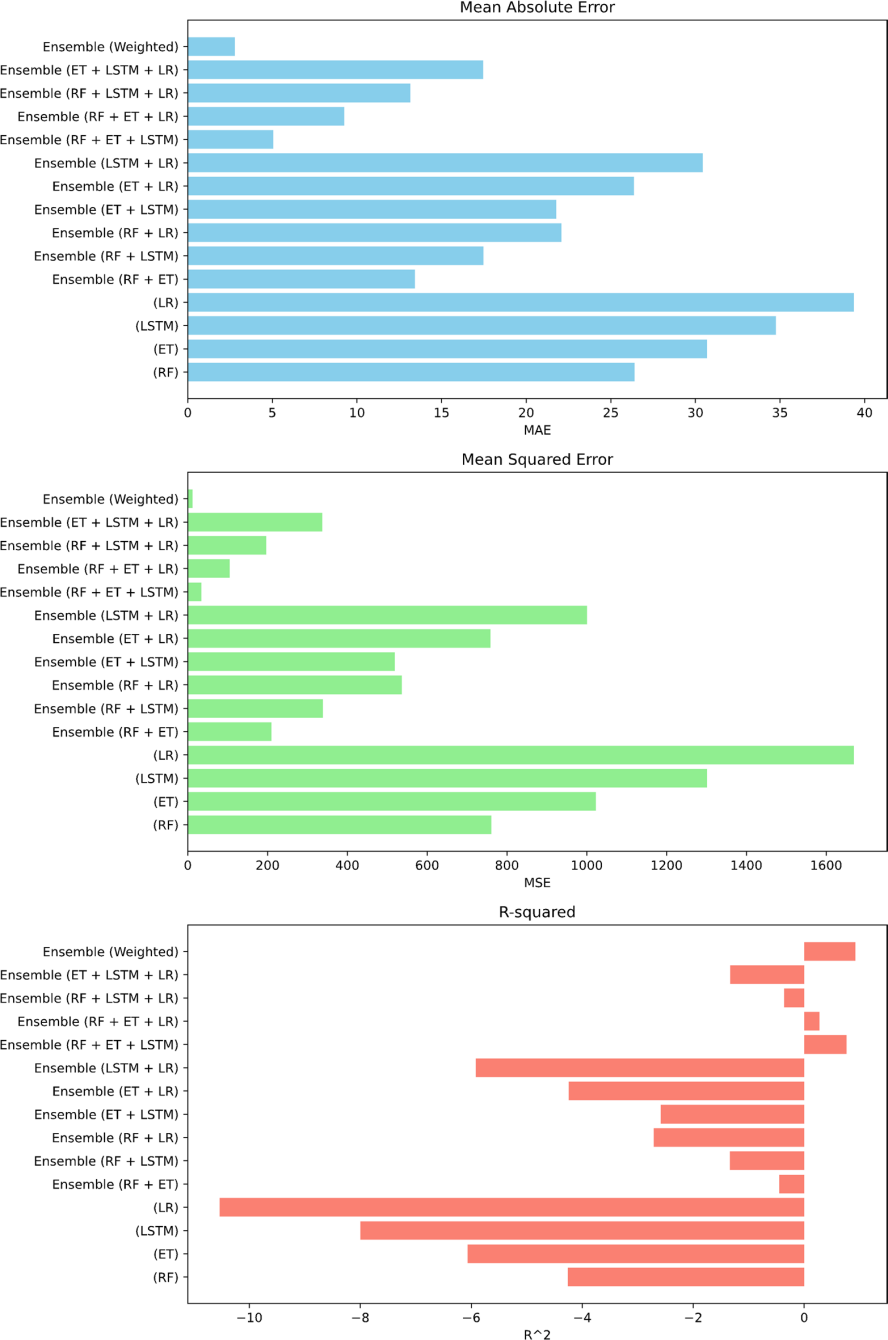
Error metrics comparison for optimum velocity: graphical overview.

The error metrics are compared in Fig. 15, and it is seen again that all four models ensembled together gives the best results with second best results being RF + ET + LSTM. This ensembling method has proven to be highly efficient, yielding strong results.

Overall, the results indicate that while Random Forest and Extra Trees consistently outperformed LSTM and Linear Regression as individual models, ensemble methods provided superior predictive performance. Among them, the four-model ensemble (RF + ET + LSTM + LR) achieved the most accurate and reliable outcomes across MAE, MSE, and R². Importantly, the 10-run average analysis confirmed the robustness of these findings, demonstrating that the ensemble models not only performed better in the best runs but also maintained consistent accuracy across multiple randomized partitions of the dataset.

### Model deployment and real-time application integration

**Fig. 16:**
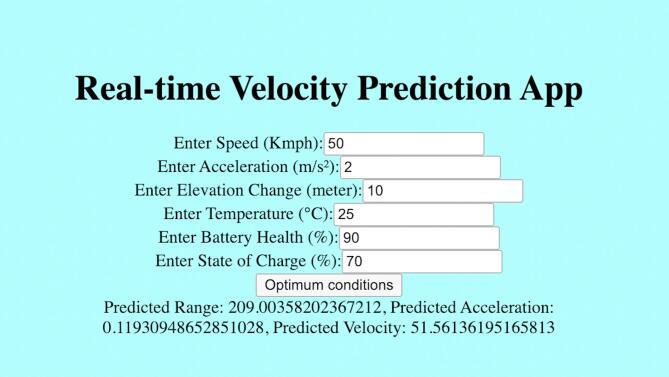
Web application integration for live model deployment.

After evaluation of all the results, the trained models are exported and used to run the web app which gives out expected results based on the input of user. Input of user can be replaced with sensor inputs and data in a hardware setting. This sums up all of the processes and gives out a graphical interface for users to understand the workings and aim of the project. These results when paired up with appropriate hardware can be used in a real time system to assist driving for a better drive experience for both the driver and the vehicle. This will also promote healthy driving habits for the user to maximise the life of their EV. A snapshot of the web page is shown in Fig. 16.

## Limitations and future scope

While the study yields encouraging results, some limitations are worth noting:

1. Absence of real-world data: the most significant limitation is the lack of real-world driving and battery telemetry. Access to such data would improve model calibration and enhance predictive performance, especially in edge cases (e.g., aggressive driving or extreme weather conditions).
2. Static feature representation: the current feature set lacks temporal granularity, which limited the effectiveness of LSTM. More context-aware input (e.g., trip segments or time-based events) could allow better use of deep learning techniques.
3. Model generalization boundaries: although the ensemble generalizes well within the scope of the dataset, further testing on varied real-world conditions is essential to validate long-range prediction stability and extrapolation accuracy.

The future recommendations can be as follows:

1. Incorporate real-world datasets: collaborating with EV manufacturers or fleet operators to obtain real operational data would significantly enhance model tuning and validation.
2. Temporal feature engineering: introduce time-series features or driving sequences to better exploit LSTM and other recurrent models.
3. Automated feature selection: apply correlation heatmaps and SHAP or permutation importance to refine feature sets and reduce redundancy.

4. Bias correction techniques: investigate calibration methods to address minor underestimation patterns in high-range predictions.
5. Model deployment evaluation: explore model performance on embedded systems or edge inference platforms for real-time range estimation scenarios.

## Conclusion

This study presents a practical and accurate approach to estimating EV range and optimal driving conditions using machine learning techniques. Among individual models, Random Forest and Extra Trees performed better than LSTM and Linear Regression. However, the ensemble of all four models consistently produced the most accurate and reliable predictions. To ensure robustness, all models were evaluated over ten independent runs with randomized data splits, and the ensemble results demonstrated consistent superiority in terms of MAE, MSE, and R² across these runs. The integration of this model into a web application demonstrates its applicability in real-time scenarios and its potential for deployment in EV systems. By providing accurate insights into range and efficient driving parameters, this work contributes to mitigating range anxiety, promoting optimal energy usage, and encouraging healthier driving habits in electric vehicle users.

## Data Availability

The data obtained through the experiments are available upon request from the corresponding author.
